# Patient and physician perspectives on treatments for low-risk prostate cancer: a qualitative study

**DOI:** 10.1186/s12885-023-11679-4

**Published:** 2023-12-05

**Authors:** Alice Guan, Eduardo J. Santiago-Rodríguez, Benjamin I. Chung, Janet K. Shim, Laura Allen, Mei-Chin Kuo, Kathie Lau, Zinnia Loya, James D. Brooks, Iona Cheng, Mindy C. DeRouen, Dominick L. Frosch, Todd Golden, John T. Leppert, Daphne Y. Lichtensztajn, Qian Lu, Debora Oh, Weiva Sieh, Michelle Wadhwa, Matthew R. Cooperberg, Peter R. Carroll, Scarlett L. Gomez, Salma Shariff-Marco

**Affiliations:** 1 Dept of Epidemiology & Biostatistics, University of California, San Francisco (UCSF), San Francisco, United States; 2 Department of Urology, Stanford University, Palo Alto, United States; 3 UCSF | Department of Social & Behavioral Sciences, San Francisco, United States; 4 Health Science Diligence Advisors, LLC, San Francisco, United States; 5 Dept of Health Disparities Research, University of Texas MD-Anderson Cancer Center, Houston, United States; 6 Dept of Population Health Science and Policy, Icahn School of Medicine at Mount Sinai, New York, United States; 7 UCSF | Department of Urology, San Francisco, United States

**Keywords:** Low-risk Prostate cancer, Treatment decision making, Active surveillance, Educational resources, Side effects, Clinical factors, Qualitative study

## Abstract

**Background:**

Patients diagnosed with low-risk prostate cancer (PCa) are confronted with a difficult decision regarding whether to undergo definitive treatment or to pursue an active surveillance protocol. This is potentially further complicated by the possibility that patients and physicians may place different value on factors that influence this decision. We conducted a qualitative investigation to better understand patient and physician perceptions of factors influencing treatment decisions for low-risk PCa.

**Methods:**

Semi-structured interviews were conducted among 43 racially and ethnically diverse patients diagnosed with low-risk PCa, who were identified through a population-based cancer registry, and 15 physicians who were selected to represent a variety of practice settings in the Greater San Francisco Bay Area.

**Results:**

Patients and physicians both described several key individual (e.g., clinical) and interpersonal (e.g., healthcare communications) factors as important for treatment decision-making. Overall, physicians’ perceptions largely mirrored patients’ perceptions. First, we observed differences in treatment preferences by age and stage of life. At older ages, there was a preference for less invasive options. However, at younger ages, we found varying opinions among both patients and physicians. Second, patients and physicians both described concerns about side effects including physical functioning and non-physical considerations. Third, we observed differences in expectations and the level of difficulty for clinical conversations based on information needs and resources between patients and physicians. Finally, we discovered that patients and physicians perceived patients’ prior knowledge and the support of family/friends as facilitators of clinical conversations.

**Conclusions:**

Our study suggests that the gap between patient and physician perceptions on the influence of clinical and communication factors on treatment decision-making is not large. The consensus we observed points to the importance of developing relevant clinical communication roadmaps as well as high quality and accessible patient education materials.

**Supplementary Information:**

The online version contains supplementary material available at 10.1186/s12885-023-11679-4.

## Background

Prostate cancer (PCa) is the most common cancer diagnosed among men in the United States (US) [1]. Advancements in screening technology and the increased use of prostate specific antigen tests have led to an increased detection of low-risk disease with good clinical prognosis [2]. Patients with low-risk PCa often face a difficult decision determining whether or not to receive definitive treatment (e.g., radical prostatectomy, radiation) or adopt active surveillance (AS). AS, the close monitoring of patients and delay of definitive treatment until signs of progression are observed, is considered the preferred management alternative for tumors that do not represent an immediate risk and might not further develop into a life-threatening disease [3, 4].

Patients with low-risk PCa following AS protocols have been found to have comparable outcomes (i.e., PCa-specific mortality, all-cause mortality) to patients receiving definitive treatment [5]. Moreover, patients on AS have the added benefit of avoiding definitive treatment’s potential side effects (e.g., sexual dysfunction, incontinence). Also, in a recent study of patients with localized PCa, those who chose AS were less likely to report treatment-related regret than those receiving definitive treatment [6]. Despite these favorable findings, the adoption of AS in the US is still low. Approximately 50% of low-risk patients chose AS, [7, 8] with observed variation across US regions, clinical settings, and physicians’ and patients’ characteristics [8–11]. Although the use of AS has increased in recent years, racial and socioeconomic disparities in the adoption and quality of AS have also been documented, [12] evidence that a widespread implementation of this option in the US has not been achieved.

It is critical to understand why adoption of well-established treatment guidelines remains low among patients with low-risk PCa. In addition to quantitative research, a call has been made encouraging qualitative approaches to better understand this issue [13, 14]. Existing research highlights the roles of physicians and patients at the time of making treatment decisions, and how their perspectives often differ [15]. Scherr et al. found that treatment decision-making among patients with low-risk PCa was dominated by physicians’ recommendations (mostly based on clinical factors), whereas patients’ preferences were overlooked [16]. Studies have also indicated how the anxiety of not treating a potentially harmful disease, fear of disease progression, and discomfort associated with procedures during AS protocols, led patients to favor definitive treatment [17].

After more than a decade since the introduction of AS to clinical guidelines, we conducted a qualitative investigation to explore both patient and physician perceptions of factors influencing treatment decision-making for low-risk PCa. Understanding what drives patients’ choices and physicians’ recommendations will help to inform future interventions to increase awareness of treatment options and acceptance of AS among racially and ethnically diverse patients with low-risk PCa. Therefore, the objective of this study was to concurrently describe patients’ and physicians’ beliefs about clinical and healthcare factors influencing treatment decision making, and to highlight similarities and differences between these two sets of perceptions.

## Methods

### Study sample

Data for the present study were obtained from a mixed methods investigation of patients’ and physicians’ perceptions of treatment decision-making for patients diagnosed with low-risk PCa. We interviewed a racially and ethnically diverse sample of PCa patients with low-risk disease, identified through a regional population-based cancer registry, and a sample of clinicians across different practice settings. We recruited eligible patients who were between the ages of 40–79 years, resided in the San Francisco Bay Area, were diagnosed with low-risk PCa within the prior 24 months, and had recently completed their initial course of disease management. While all men in the sample had PCa diagnoses that were eligible for AS, sampling was agnostic to treatment that was received so that we could capture the range of perspectives associated with decision making. To ensure that a racially and ethnically diverse sample of patients was included, we intentionally recruited participants from different groups including Black, Chinese, Filipino, other Asian American (i.e., Asian Indian, Japanese, South Asian, and those who did not further specify their Asian ethnic group), Hispanic/Latino, and White individuals through the Greater Bay Area Cancer Registry. Eligible physicians included those who treated patients with low-risk PCa in the San Francisco Bay Area, and were recruited through snowball and purposive sampling, selected to represent a variety of practice settings (e.g., academic medical centers, community medical centers).

### Data collection

The semi-structured interview guide for patients was designed to capture complex and multidimensional perspectives on factors relevant to the treatment decision-making process. Patients were asked to recount their experiences around diagnosis, decision-making, and following treatment. All interviews were conducted in participants’ preferred language and those conducted in Spanish or Chinese were transcribed into English. Transcripts and translations were reviewed for accuracy by interviewers who also were involved in the translation and back translation of all study materials. Qualitative interviews for physicians included questions about physicians’ perceptions about patients’ treatment preferences, as shared through two case examples – a challenging case and an easy case. Both interview guides are included in the supplemental materials. The physician interview questions were developed collaboratively with the physician co-investigators and a medical sociologist, while the patient interview guides incorporated input from epidemiologists, a psycho-oncologist, physicians, a medical sociologist, and preliminary insights from patient surveys. Our interviews were also intended to be semi-structured, thus balancing questions guided by preliminary data and our study team’s experience and expertise, as well including questions that were more open-ended to allow for emergent, unanticipated data.

### Data analysis

We conducted thematic analysis for both patient and physician interviews in Dedoose, a qualitative data analysis software platform. Analysis of patient interviews have been described in detail in a prior manuscript which focused specifically on examining racial and cultural factors driving decision making, and a similar approach was used to analyze physician interviews [18]. In brief, for both sets of interviews, at least two independent investigators were trained to code the qualitative data in a training set of interviews using an initial codebook that was developed to reflect core themes in the interview guides. Once reliability of coding was satisfactory (i.e., a majority of the transcript was coded similarly across coders, and strategies for handling specific coding scenarios were agreed upon), the full set of transcripts was assigned to each independent coder. Three rounds of coding and discussion resulted in a final set of 29 codes for the patient interviews, and 25 codes for the physician interviews, which were used to analyze the full set of transcripts. The subset of codes that were analyzed for this paper are presented in Table 1. For this study, we sought to understand and compare how patients and physicians describe the role of clinical factors, concerns about side effects, and patient’s general health profiles (including age and comorbidities) in treatment decision-making. As such, we initially focused our qualitative analysis on reviewing text segments with codes related to these domains in each set of interviews. Several additional themes emerged related to information, communication, and patient experiences with physicians and healthcare. Thus, we expanded our analysis to include codes in these domains. To compare and contrast patients’ and physicians’ views, we provide a simultaneous presentation of these findings from patient and physician transcripts. Data is presented following the Standards for Reporting Qualitative Research (SRQR) [19]. The SRQR checklist can be found in the supplemental materials.

**Table 1:** Analytic codebook for patient and physician qualitative data, organized by theme

	Codes reviewed from Patient Interviews	Codes reviewed from Physician Interviews
Clinical factors	Clinical factors Comorbidities/prior experience with illness Second opinion Age** Cancer-related anxiety	Age Active surveillance risk Side effects Clinical factors Genetics/genetic testing General health
Healthcare factors	Health insurance Feelings/experiences with healthcare system Feelings/experiences with MD Knowledge/information gathering	Physician support Patient-centered communication Empowered patient Patient reaction Patient refusing recommendation Patient education Informational materials

** In patient interviews, excerpts related to “age” were identified through lexical search of the following terms: age, young*, old*, and year* to identify statements related to this concept

## Results

### Sample characteristics

A description of the characteristics of patients is presented in Table 2. Of the 43 patients, 30% were Asian American (5 Chinese, 3 Filipino, and 5 Other Asian American, including Asian Indian, Japanese, South Asian, Vietnamese, and one participant who did not specify their ethnic group), 23% were Black, 23% were Hispanic/Latino, and 23% were White. Approximately half (44%) of patients were diagnosed between age 50–59. Most patients were US-born (63%), married (79%), completed college or more (54%), employed (56%), had annual household income of ≥$100,000 (47%%), and had private health insurance (61%). Less than half (44%) of the sample opted for AS to manage their PCa. A description of the characteristics of the 15 participating physicians is presented in Table 3. Of the 15 physicians, the mean age was 44.6 years (SD 8.0), with an average of 12 years (SD 8) of practicing medicine. The majority were male (80%) and White (53%), and approximately half worked in community settings (47%). Most of the physicians in our sample were urologists, though 1 respondent specialized in trauma recovery.

**Table 2:** Demographic characteristics of patients with low-risk prostate cancer recruited from the Greater San Francisco Bay Area between 2018–2019 (N = 43)

Characteristic	N (%) or Mean, SD
Race/Ethnicity	
Chinese American	5 (11.6)
Filipino or other Asian American*	8 (18.6)
Black	10 (23.2)
Hispanic/Latino	10 (23.2)
White	10 (23.2)
Age at diagnosis	
50 to 59	19 (44.2)
60 to 69	15 (34.9)
70 and older	9 (20.6)
Born in the United States	27 (62.8)
Marital status	
Currently married or living with a partner as married	34 (79.1)
Never married, separated, or divorced	9 (20.9)
Highest level of education completed	
High school/GED or less	7 (16.3)
Some college	13 (30.2)
College graduate	11 (25.6)
Post-college graduate	12 (27.9)
Employment Status	
Employed	24 (55.8)
Unemployed (includes welfare and disability) or self-employed	5 (11.6)
Retired	14 (32.6)
Household size	2.7, 1.4
Total household income	
Less than $100,000	15 (34.9)
$100,000 to $149,999	7 (16.3)
$150,000 or more	16 (30.2)
Don’t know/Refused	5 (11.6)
Health Insurance	
Medi-Cal or Medicare	10 (23.2)
Medicare and other (including Medi-Cal, Private Insurance, and VA)	7 (16.3)
Private Insurance	26 (60.5)
Treatment received	
Active surveillance	19 (44.2)
Active treatment (e.g., radiation, surgery)	24 (55.8)

*3 patients self-identified as Filipino American and 5 self-identified as another Asian American group. Survey options for Asian American groups included: Chinese, Filipinos, and Other Asians. Specific Asian American subgroups specified for those who selected “other Asian American” included Japanese, South Asian, and Vietnamese

**Table 3:** Demographic and practice characteristics of a sample of physicians treating low-risk prostate cancer patients in the Greater San Francisco Bay Area (N = 15)

Characteristic	N (%) or Mean, SD
Age	44.6, 8.0
Gender	
Male	12 (80.0)
Female	3 (20.0)
Race	
Asian American	7 (46.7)
White	8 (53.3)
Years practicing	12.6, 9.2
Practice setting	
Academic	3 (20.0)
Community	7 (46.7)
Solo/group practice	4 (26.7)
VA	1 (6.7)

Main findings of the study are summarized in Table 4 and described below. Generally, our analysis did not reveal meaningful racial or ethnic differences in descriptions of clinical factors associated with decision making.

**Table 4:** Factors influencing treatment decisions among low-risk prostate cancer patients in the Greater San Francisco Bay Area according to patients and physicians

Themes	Description
Age and stage of life	• At *older ages*, preference for avoiding definitive treatment options. Main factors considered: o More comorbidities o Higher risk of death from other causes o Shorter life expectancy • At *younger ages*, opinions varied. Factors for favoring definitive treatment options: o Healthier status to handle complications and having a faster recovery o Longer life expectancy o Quick and definitive action versus longer duration of active surveillance Factors for favoring other options: o Delay of definitive treatment at the possibility of mild disease o Interest in preserving body functions
Side effects	• Fear of side effects affecting well-being and physical functioning o Hair loss, nausea, erectile dysfunction, incontinence • Experiences of side effects during past medical encounters o Other procedures- complications • Family opinions o Disregarding patients’ concerns about side effects and urging definitive treatments as the best options to ensure they live longer
Facilitators of meaningful clinical conversations about treatment	• Differences in expectations and the level of difficulty for clinical conversations based on information needs and resources between patients and physicians o Physicians perceived that higher patient medical literacy made conversations easier and more informative o Perceptions of the role of health literacy less prominent among patients • Prior knowledge and the support of family/friends o Friends/family sometimes advocated for more aggressive treatments o Importance of support for patient’s decisions about treatment

### Theme 1: age and stage of life

Patients and physicians shared a consensus that older patients preferred AS, and that younger patients expressed a wider range of treatment preferences.

We observed agreement between patients and physicians on how older patients (late 60s and beyond) with low-risk PCa approached treatment decisions. In general, these patients described a preference for less invasive options irrespective of health status. For instance, a patient contrasted how his current stage of life influenced his treatment decision with what he would have done at a younger age. *“At my age and where I am – I’m semi-retired. There was not a driving need to cure this at all costs… Had I been younger, I think I might have accepted a more aggressive approach.”* (study ID: 50014). Another patient mentioned comorbidities at older ages and higher risk of death from other causes as drivers of treatment decisions. Specifically, his belief that other health conditions posed a greater risk than the indolent malignancy of low-risk PCa led him to favor AS. *“If their cancer is low grade and they know about positive calls…, then they should go on active surveillance…Why go through something that is so that dramatic when you know your high blood pressure, weight, could kill you before that? So, you got to consider, your health, your age and all this other stuff.”* (50102). Similarly, physicians observed that older patients with low-risk PCa favored less radical options. One physician shared that it was common in their practice: *“I’ve had several patients who are in their late sixties, very sexually active, very healthy overall and they have low-volume Gleason 3 + 4, and then I do genomic analysis and it is still borderline and they’ll do almost anything to avoid having surgery or radiation.”* (6011). Another physician portrayed the case of a patient discussing different options and how considerations around age and life expectancy helped him to determine the preferred choice:

“*He’s older (70s), he doesn’t have a long-life expectancy, he’s a low-risk disease patient, and approach it just the way I spoke before. I talked to him about the risk stratification, the Gleason scoring, and all the potential treatment options. And then, for him, we really talk about that active surveillance for him in terms of all of his other competing comorbidities and medical disease and life expectancy, just makes the most sense.”* (6004).

In contrast, patients and physicians both described that younger patients (50s-low 60s) had more variation in their treatment preferences. Some younger patients preferred definitive treatments because they were healthy, had less comorbidities, and could deal with complications at their current age more easily than at an older age. A patient mentioned: *“A healthy person like me, only 60, I take action, not active surveillance. Because surgery is not easy, … the older you are, the harder recovery from incontinence. Your muscle’s weak, everything weaker, you know what I mean?”* (50215). Physicians also described younger patients who preferred definitive treatments, specifically expressing concerns about the long duration of an AS protocol coupled with beliefs that immediate treatment would help to prolong their lives: *“For this patient, I think they come at it very similarly to us in that, they’re hesitant about active surveillance at that age. Because, they see the time horizon being as long as 25, 30 years, right? So, I think because of that, they’re also thinking about their kids and being around for them.” (6009).*

Other younger patients preferred less invasive treatments. One patient recalled, *“I just thought it was less invasive and I thought I was young enough where I didn’t have to do a full removal, like I still got time left. I don’t think I want to do that right now and I wasn’t really into doing the everyday radiation thing, so I chose to do the brachytherapy.”* (50047). This patient’s decision-making was influenced by his perception that he could have many years to live with a potentially mild disease and could delay more aggressive treatment options for the future. These perspectives were also observed in physician interviews, with one physician stating, *“In general, younger patients with low grade disease tend to favor either active surveillance or possible brachytherapy. They tend to shy away from radical prostatectomy in my practice.”* (6005). Another physician echoed this point of view and emphasized the role of patients’ educational attainment, lifestyle, age, and social obligations in treatment decision-making: “*So those are usually the well-educated, kind of younger groups who really want to preserve their erectile function as well as urinary function.”* This physician then added an example of one patient:

*“When I made the recommendation of active surveillance, he felt that it was a really good and appropriate recommendation, understanding that this would help preserve his erectile function as well as his functional mobility. He didn’t really want to take time from work, off of work. He didn’t foresee wanting to lose time in his triathlon training and didn’t want to go through the recovery process that a surgery would entail.”* (6014).

### Theme 2: side effects

There was widespread agreement between patients and physicians that side effects were very important for treatment decision-making. We uncovered three distinct ways in which side effects influenced decision making: fear of side effects, the influence of side effects from past medical encounters, and the intersection of family opinions with side effects. Specifically, patient considerations of side effects were often trumped by the opinions of family members, who often desire survival above all else and equate the aggressiveness of a treatment with better survival.

The most common side effects mentioned by patients were the immediate disruptions to physical functioning following invasive treatment. One of the patients described: *“I was concerned that prostate surgery or radiation would have side effects; incontinence, impotence, things like that. And I wanted to avoid those if I could.”* (50014). Patients also described how concerns about side effects which were more distal to treatment influenced their treatment decision-making. As one patient said, *“Rumor has it that chemotherapy has serious side effects. People said so. I am not really sure about it. Hair loss, loss of appetite, nausea, I don’t want that.”* (50158). Though the side effects might not be long lasting, his concerns about them were unpleasant enough to make him hesitate to receive these treatments. Patients also talked about the possibility of presenting long-term side effects that could even evolve over time. One patient shared:

*“So I went to see another doctor that’s doing radioactive material implants. … And I came to discover that, for those treatments, the side effects were maybe minimal at the beginning, but it gets worse with time, like receiving radiation, and just start with feeling weird at the implanted area. And then maybe some discomfort as well. When the discomfort was minor, and then the sexual ability is almost unaffected. But then as time goes by longer, when the treatment has worn out, then the bad parts start to show up, and it’s not a good option to take.”* (50170).

These comments reflect patients’ reluctance to procedures that could represent an additional burden to their health, and a preference for other potentially less detrimental treatment options. Physicians observed similar concerns about side effects for their patients. As one physician mentioned, *“Patients are very reluctant to hear about the urinary and erectile misclaim, irritability with radiation as well as the incontinence with surgery.”* (6014). Based on their own voices and on physicians’ perceptions, patients with low-risk PCa were aware and very cautious of the potential side effects they could face if receiving definitive treatment.

In addition to fears about future side effects, patients described how side effects from past treatments influenced their current decision-making. For example, patients talked about prior experiences within the healthcare system, especially procedures that had harmful consequences on their health. A patient commented:

*“I had prostate surgery and it was because I was having, obviously, a lot of urinary infections. And I did not have the traditional method. I had the method that is done, I think, with laser and like that, it’s supposed to produce less waiting. As a result of it, I had numerous infections afterwards, and he [doctor] said something about I had excessive bleeding. For a surgery that was supposed to be minimal bleeding, it was a lot of bleeding, and I had to wear the apparatus for the urination and the blood for a lot longer than you normally wear it. I think I had that on for six weeks. I had three infections simultaneously on top of each other. So, it wasn’t successful, in my opinion…. So, I said, Great, ‘cause I really don’t want to go through another surgery and I’m still having the problems from the last surgery.”* (50018).

Physicians also highlighted how patients’ treatment decision-making was influenced by negative experiences with previous medical procedures. One physician mentioned, *“Now, importantly to note, the patient was very against re-biopsy. He did not enjoy his prior experience.”* (6014). In this case, the physician did not describe concerns about side effects related to a radical treatment option. Rather, the physician reflected on a patient concern about “side effects” of future biopsies, a key component of AS.

In addition to concerns about physical side effects, many participants expressed worry about less tangible impacts of their treatment decision. Patients described their situations balancing their fear to experiencing painful circumstances on their bodies with family member’s desires for survival above any other consideration. A patient stated: *“She [partner] didn’t care about the side effects. She wanted me alive, she didn’t care about side effects.”* (50102). Another patient shared a similar experience, in which the partner believed surgery was the best alternative after a PCa diagnosis, despite of being low-risk: *“And so I came prepared, and my wife pushed me to receive the operation. And I tried not to let her affect me… She wanted me to survive. She didn’t want other things.”* (50170).

### Theme 3: facilitators of meaningful clinical conversations about treatment

Finally, both physicians and patients described factors that made conversations about treatment decisions easier, though we observed minor differences in their descriptions of these factors. These factors included patient self-education and knowledge about PCa prior to the clinical encounter and the supportive role of family and friends.

Both physicians and patients mentioned the helpfulness of prior information and knowledge about PCa in facilitating conversations about treatment. Among physicians, there was broad sweeping sentiment that conversations about treatment were easier when patients were proactive about information gathering. Such sentiments from physicians included:

*“I would say that the most challenging patients are the ones that don’t come in with a lot of information, where you’re doing a lot of the education and where they turn right back to you and say, ‘Well, what do you want me to do?’” (6001) and “[The patient] pre-educated himself, and it was the easiest conversation in the world because he was pre-educated.” (6008).*

These physicians’ comments both suggest that lack of access to information led to more frustrating and challenging clinical conversations. Another physician noted:

*“I don’t feel like we’ve had a two-way communication. I feel like I lectured and because of the lack of full medical literacy that this patient really didn’t’ make such an informed – I mean, I tried to inform him – but I’m not sure that he really understood.” (6004).*

This physician’s description illustrates how a lack of information from patients not only hindered the clinical conversation, but also obfuscated consequences of treatment for the patient. Altogether, the importance of patient education and information gathering was abundantly observed across physician interviews. Although this sentiment was also expressed in the patient interviews, it was less common than was found in the physician interviews. One patient mentioned:

*“[My doctor] told me it was a Gleason score of 3 plus 3 equals 6. I don’t think we had a detailed discussion at the time as to how that fit into other types of cancer. But the research that I’ve done, subsequent to that, makes it very clear the scale was 6 to 10.” (50012).*

Though not explicit, this patient’s recollection of this conversation makes clear that prior knowledge and information gathering would have allowed for a more engaged conversation about his Gleason score.

Additionally, though both physicians and patients described a range of information sources (e.g., web-based, support groups), there was consensus about the importance of family and friends in supporting patients through the decision-making process. Many patients described the input of family and friends as bolstering physician recommendations. For instance, in describing his thought process as he was deciding between AS and definitive treatment, one participant stated:

“*I was leaning… towards [active surveillance] until the second genomic testing results came back… It was then declared as more aggressive than the low-risk scenario, sort of borderline… Ultimately, my doctor said, “I’d recommend taking active measures” … That was also backed by another family friend who also factored in my age and said, ‘If you were my husband, I’d bully you to get something.’ So, I chose.*” (50160).

For this patient, both his friend and physician advocating for treatment compelled him to get definitive treatment. The importance of consensus between physician recommendation and family advice was also noted by physicians. For instance, when asked how his patient felt about an AS recommendation, one physician stated:

*“He was thrilled about it… But it is really important to have the family members on the same page, because I think having your wife telling you every day that you should get treated can probably wear on guys and grind them down.” (6015).*

This physician’s comments were echoed by others in the sample and represents an understanding of the importance of involving family members in the treatment decision-making process. Physicians also noted instances in which family members forced certain treatment options, usually more aggressive ones. This physician recalled himself saying to a patient’s daughter:

*“Yes, yes, cancer, but this cancer he could have never had any of this done, and he will probably die in 10–12 years naturally, and this won’t kill him. But no one will listen to me on this and everyone wants to know: what else can we do other than do nothing? So all this is going through the daughter, and then to the patient. So the conversation instead of being a 15 minute conversation, is a 30 plus minute conversation where I’m not even sure if the patient’s getting all the information from his daughter, which is really frustrating.” (6008).*

## Discussion

In this study, we sought to compare patient and physician perceptions of clinical and healthcare factors that were relevant to treatment decision-making for patients with low-risk PCa. Using data from a population-based racially and ethnically diverse group of low-risk PCa patients and from physicians across a variety of practice settings, we found that there was substantial consensus between patient and physician descriptions of the influence of age, side effects and comorbidities, and clinical conversations on a patient’s treatment decision-making. Our analysis did not reveal meaningful racial or ethnic differences in the factors that we described, emphasizing the shared impact of clinical and healthcare factors in the context of low-risk PCa treatment in our sample. Areas of concordance and gaps related to these factors could point to promising strategies and approaches for higher quality, patient-oriented care.

Patients and physicians both agreed that older patients were more likely to prefer AS, which is congruent with quantitative studies using national, longitudinal data of men with PCa reporting that frequency of selecting AS increased with age [20]. Similarly, patients and physicians found more varied treatment preferences among younger patients, which could potentially reflect the broad range of views that physicians have about recommending treatment for low-risk PCa in this age group. For example, a prior study described that younger patients are often counseled to select definitive treatment options because they have fewer comorbidities [21]. Similarly, physicians in another qualitative study expressed hesitance in recommending AS for younger patients, a sentiment that was more pronounced among academic urologists [22]. In fact, the use of AS to manage low-risk PCa has been found to widely vary at both the physician and practice level, [9] further bolstering the large variation in younger patient’s perspectives about treatment. Additionally, while both patients and physicians in our study described a keen awareness of the influence of side effects – from fears of side effects on health and other domains of life to fears stemming from past experiences with side effects – they also described how these fears were often trumped by family members opinions about treatment. We found that younger and older patients gave similar descriptions of the importance of the opinions of family in their PCa treatment decision making, indicating that education and interventions targeting family members could be a promising area of future research.

Both patients and physicians in this study described two key facilitators of meaningful clinical conversations about PCa treatment. These facilitators included patients’ prior knowledge and information about PCa, as well as the supportive role of friends and family, both of which could be meaningful points of engagement for treatment decision-making. A prior study found that patients with low-risk PCa are not provided with sufficient information to make an informed treatment decision [23]. This lack of access to educational resources about PCa has been found to cause an over-reliance on the opinions of physicians, family, and friends in the treatment decision-making process [24, 25]. However, there are documented differences in preferences for PCa care between physicians and patients, [26] and physician specialty has been found to be strongly predictive of their treatment preferences and recommendations [27]. Therefore, our findings suggest that interventions aimed toward providing education to patients and their families can help bridge gaps in clinical conversations about treatment and further facilitate patient self-empowerment. For example, computer-based education programs have been associated with improvements in decisional support during diagnosis and treatment [28, 29]. Unfortunately, many existing patient materials are inadequate. For instance, a cross-sectional review of publicly available patient education materials for the treatment of early-stage PCa found that 92% of materials did not provide descriptions of all guideline recommended treatments (i.e., AS, surgery, and radiation) [30]. Additionally, despite the high-quality and informative patient education materials that do exist, [31–33] patient knowledge of and access to the appropriate materials is inconsistent. The amount of informational support patients received within clinical settings has been found to be similarly inadequate, as a recent scoping review found that patients undergoing AS to manage their PCa reported that information and communication received during follow-up was only cursory [34]. This suggests that though patient education can promote patient self-sufficiency and more meaningful conversations with clinicians regarding treatment options, there is a need for high quality, reliable, and comprehensive content that is accessible to all patients.

There are several limitations of this study. Patients in this study had already decided on their first course of disease management. Therefore, our findings may not capture feelings and experiences of participants during the treatment decision-making process. Our findings also may not be generalizable to individuals outside the Bay Area or necessarily reflect the perspectives of patients from lower socioeconomic backgrounds. However, the primary objective of qualitative research is not to achieve generalizability but rather to comprehensively describe the diverse range of perspectives present in the studied population. Additionally, while our patient sample is racially and ethnically diverse, all physicians in the sample were either White or Asian American. There could be substantial variation in the perception of factors we described in physicians of other racial and ethnic groups. Also, because physicians were recruited through snowball and purposive sampling strategies to capture a variety of practice settings, findings could be reflective of all conventional approaches to treatment and patient communication that may exist. Furthermore, this study was conducted with patients and physicians in the Greater San Francisco Bay Area, where adoption of AS is reflective of local academic efforts. Finally, although we simultaneously described the perspectives of physicians and patients, the two participant groups were unlinked, and therefore, it is possible that there is limited geographic and demographic overlap. It may be informative in future studies to evaluate how perspectives differ within clinician and patient dyads.

In conclusion, our study suggests that patients and physicians generally agreed on the influence of clinical and communication factors on treatment decision-making. In other words, patients and physicians in this study did not describe decision-making through vastly different worldviews, nor did they have distinctive criteria lists for treatment decisions. Additionally, while there were minor differences in description of these factors, the consensus that we observed suggests several potential avenues for engagement and intervention with patients. First, our findings point to the need to support efforts to develop clinical roadmaps for physicians and institutions serving patients with low-risk PCa from diverse populations [9, 35]. Additionally, a potential area for future research could be to better understand the role of family members in treatment decision-making. Furthermore, our findings suggest that the promotion and development of high quality, comprehensive, and, perhaps most importantly, accessible and culturally-tailored education materials for patients and their families can help to bridge the gap in clinical conversations about treatment of low-risk PCa. Lastly, considering the immense impact that changes in clinical guidelines and screening recommendations have had on community uptake of AS, further adoption of active surveillance especially in community settings may require policy-level changes.

### Electronic supplementary material

Below is the link to the electronic supplementary material.

Supplementary Material 1

## Data Availability

The data that support the findings of this study are available on reasonable request from the principal investigator of the study, SLG. The data are not publicly available as it contains information that could compromise the privacy of research participants.

## Abbreviations

AS: Active Surveillance
PCa: Prostate Cancer
US: United States
