# Supplementary material for: Patient and physician perspectives on treatments for low-risk prostate cancer: a qualitative study
Source: BMC Cancer. 2023 Dec 5;23:1191. doi: 10.1186/s12885-023-11679-4 (PMC10696696; doi:10.1186/s12885-023-11679-4)
Supplement: Supplementary file 1 — Supplementary Material 1 [file 12885_2023_11679_MOESM1_ESM.docx]

**SUPPLEMENTAL MATERIALS**

**Patient and physician perspectives on treatments for low-risk prostate cancer: A qualitative study**

**Patient Interview Guide**

The following is the topic guide that was used to conduct semi-structured interviews with patients with low-risk prostate cancer.

- I would like to start by having you describe the “story” of your prostate cancer experience. It would be great if you could start with how you found out that you had prostate cancer.
- As a [specify race or ethnicity] man, what does it mean to you to have prostate cancer?
- What were your thoughts and feelings when you discovered that you have prostate cancer?
- How did your family react to your diagnosis?
- Tell me about the discussion you had with your doctor about your treatment options.
- Did your doctor tell you about different types of prostate cancer?
- Do you remember what he/she said about your prostate cancer?
- Did any of your doctors recommend a particular treatment option?
  - If yes, did you agree with the recommendation?
  - If no, did you feel comfortable expressing disagreement with the recommendation?
  - If not comfortable, what got in the way?
  - If AS not mentioned: You have not brought up AS during the discussion. What, if anything, did your doctor have to say about AS for you? *Probe about pros and cons*.

*If participant asks, “what is active surveillance?”:* Because prostate cancer often grows very slowly, some men might hear from their doctor that they may never need treatment for their prostate cancer. Instead, their doctors may recommend an approach known as active surveillance or sometimes called watchful waiting, observation, or expectant management. Active surveillance means your doctor would monitor you closely by seeing you regularly at office visits and getting tests, but not treating your cancer otherwise. Did your doctor talk about this approach for you?

- What things were important to you when thinking about treatment? Or, please describe how your prostate treatment decision was made.

What personal factors led to the decision about the treatment you received?

- Family history
- Prior experience with illness
- Prior experience with health care system
- Your personal religious beliefs
- Your heritage and family background

What relationship factors led to the decision about the treatment you received?

- Your relationship with your intimate partner
- Your partner’s beliefs about prostate cancer
- Your partner’s expectation about treatment outcomes
- The way you discuss important things with your partner
- Your partner’s concerns about you
- Did your partner have other preferences for your prostate cancer treatment? [If YES] What were they/can you explain?

What health care factors led to the decision about the treatment you received?

- Your relationship with your doctor
- Your medical insurance
- What you know about prostate cancer
- Your beliefs in honesty and fairness of the health care system
- Your trust in your doctors
- Any lessons learned you would like to share with others who might be trying to decide about treatment for their prostate cancer, including active surveillance?
- Do you have other thoughts that we have not covered?

**Physician Interview Guide**

We are conducting these one-on-one interviews in order to really get a sense of what the conversations about prostate cancer diagnosis and treatment are like with your low-risk patients, and how they understand the information you provide to them. We’d also like to know about the types of patients you see – where they come from, what sort of sociodemographic characteristics do they have, whether your practice is primarily referral, etc.

To that end, we’d like you to walk us through your interactions with a couple low-risk prostate cancer patients you treated or consulted with. I am especially interested to hear about your interactions with racial/ethnic minority patients. We’d like to hear about one “easy” case and one “challenging” case, in terms of conveying information about their prostate cancer and treatment options. Starting with the most challenging case, please tell me how the patient was diagnosed, what their clinical presentation looked like, maybe talk about how well you know the patient, who came with them to the appointment(s), etc.

Let provider tell the story of an example of a “challenging” low-risk patient. Then ask him/her to tell you about an “easy” low-risk patient.

Probes:

- What were the options you presented?
- Did he ask questions about the options? (if appropriate) Did any family members/friends ask questions?
- What factors did you think were most important, with this particular patient, in making the recommendation that you did? [comorbidities, functional status, compliance history, age, insurance, marital status, employment status, family history of prostate cancer and breast cancer)
- How did the patient feel about your recommendation?
- Have you found that certain types of patients tend to favor a certain type of treatment? Are there patient characteristics that tend to predict treatment choice (e.g. personality/anxiety, educational level)?
- Does your practice provide any informational materials? What are they? Do they cover pros and cons of various treatment options?
- What is your perception of active surveillance? How much were you exposed to active surveillance during training?
- In your view, what is most important to you about communicating with your patients? [After this, then ask] There has been a lot of discussion recently about “patient-centered communication” — what does that mean to you?
- Any lessons learned you would like to share with us about talking to low-risk prostate cancer patients about their treatment options?
- Is there anything else you want to tell us?
